# A fast statistical significance test for baseline correction and comparative analysis in phase locking

**DOI:** 10.3389/fninf.2013.00003

**Published:** 2013-02-15

**Authors:** Kunjan D. Rana, Lucia M. Vaina, Matti S. Hämäläinen

**Affiliations:** ^1^Brain and Vision Research Laboratory, Department of Biomedical Engineering, Boston UniversityBoston, MA, USA; ^2^Athinoula A. Martinos Center for Biomedical Imaging, Massachusetts General HospitalCharlestown, MA, USA; ^3^Department of Neurology, Harvard Medical School, Massachusetts General HospitalBoston, MA, USA

**Keywords:** MEG, phase locking, oscillation, cross-talk, circular statistics

## Abstract

Human perception, cognition, and action are supported by a complex network of interconnected brain regions. There is an increasing interest in measuring and characterizing these networks as a function of time and frequency, and inter-areal phase locking is often used to reveal these networks. This measure assesses the consistency of phase angles between the electrophysiological activity in two areas at a specific time and frequency. Non-invasively, the signals from which phase locking is computed can be measured with magnetoencephalography (MEG) and electroencephalography (EEG). However, due to the lack of spatial specificity of reconstructed source signals in MEG and EEG, inter-areal phase locking may be confounded by false positives resulting from crosstalk. Traditional phase locking estimates assume that no phase locking exists when the distribution of phase angles is uniform. However, this conjecture is not true when crosstalk is present. We propose a novel method to improve the reliability of the phase-locking measure by sampling phase angles from a baseline, such as from a prestimulus period or from resting-state data, and by contrasting this distribution against one observed during the time period of interest.

## Introduction

Indices of consistent phase differences at a particular time and frequency are commonly used to assess coherence relationships between two signals. It is calculated by first computing the time-frequency representations of a pair of time-varying signals. This may be accomplished through a variety of means, including wavelet filtering and by applying the Hilbert transform. Thereafter, the phase difference at the corresponding time and frequency points between the two time courses is computed on a trial-by-trial basis to test for repeatability. In magnetoencephalography (MEG) and electroencephalography (EEG), phase locking is evaluated either between two sensor signals, between two estimated source time courses, or between one sensor or source time course and an external reference signal such as the electromyogram (EMG) (Tallon-Baudry et al., 2001; Lin et al., 2004; Schoffelen and Gross, 2009).

Constant phase differences between activation time courses can be attributed to functional connectivity. In particular, two areas whose estimated source time courses have a consistent phase difference are likely communicate with each other through some pathway or may be jointly affected by a third source.

If the phase relationship is not fixed, the difference of the phase angles will be random with no bias toward a specific angle. Therefore, under the null hypothesis of no phase consistency, the angles will have a uniform circular distribution. However, in reality, various unavoidable confounds in data processing may lead to false consistent phase differences.

Non-invasive recordings of human electrophysiological activity can be obtained with EEG and MEG. However, mapping the MEG/EEG sensor signals back onto the cortex is an ill-posed inverse problem (Hämäläinen et al., 1993) and, therefore, appropriate constraints and regularization need to be applied to render the solution unique and stable. One common solution known as the minimum-norm estimate (MNE), finds a smooth, distributed current distribution with minimum L2 norm constraint to reproduce the measured data (Hämäläinen and Ilmoniemi, 1994). With help of high-resolution MRI data, it is possible to reconstruct the geometry of the individual cortical surface (Dale et al., 1999; Fischl et al., 1999a,b, 2001) and use this information to create location and orientation constraints for source modeling (Dale and Sereno, 1993; Dale et al., 2000; Lin et al., 2006).

However, due to its distributed nature, the MNE will exhibit strong false spatial correlations due to spread and cross talk in the estimates, which often lead to such false positives (Schoffelen and Gross, 2009). Beamforming methods (Van Veen et al., 1997; Robinson and Vrba, 1999; Hui et al., 2010; Rana et al., 2011), designed for spatial filtering of data, help to reduce correlations, but will still tend to map the same sensors, with variable weights, onto multiple ROIs, and this can also lead to false positives in phase locking.

With direct measurements of neuronal activity through single-unit, multi-unit, or local field potential (LFP) recordings spaced apart to avoid strong correlations, phase locking methods can be applied without concerns of confounds present in MEG/EEG source modeling. However, the resulting data will only allow for inferring phase locking amongst a small set of neurons instead of between functional brain areas. Thus, invasive recordings only solve the issue of crosstalk but cannot be efficiently performed across the cortex. In addition, direct invasive recordings are very rarely conducted in humans and, when they are, are used in clinical cases such as in epileptic patients (Gloor, 1975; Keene et al., 2000).

In this methodological paper, we propose a novel, fast method with which the distribution of phase angle differences between two areas are compared to an arbitrary null distribution, e.g., to the distribution of phase angles in the absence of the stimulus, via a non-parametric phase angle method with asymptotic bounds, producing significance levels with minimal computational cost. We will describe the traditional phase locking method and compare it to our new method, Uniform Scores Test for Phase Locking (USTPL). In addition, we shall discuss two recently introduced methods that also provide a means for baseline correction: the Phase Lag Index (PLI) and the Phase Bifurcation Index (PBI). We will show how non-parametric statistical methods applied directly to the phase angle differences result in an accurate significance test as opposed to using a parametric test that falsely assumes a uniform null distribution.

## Methods

The first section here discusses the nature of the simulated data and how data is mapped onto the brain. The second section elaborates on the four methods that are the focus of the comparison: traditional phase locking, our proposed method USTPL, as well as PLI and PBI.

### Data simulation

#### Brain surface and region of interest (ROI) selection for data simulation

**Figure 1:**
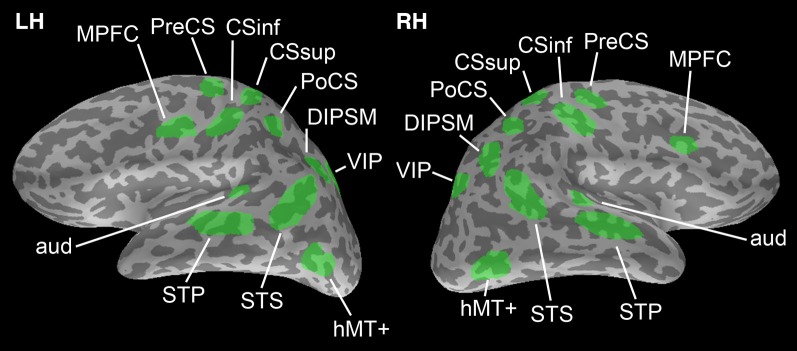
**Regions of interest chosen from which to generate simulation data.** ROIs are adapted from Vaina et al. (2010); Calabro et al. (2011). There is no overlap between ROIs to avoid any issues in computing phase locking connectivity between them.

The brain surface used to produce simulations is from a healthy male subject, age 23 at time of collection. Structural MRI scans were acquired using an 8-channel phase-array head coil in a 3T scanner (Siemens-Trio, Erlagen, Germany). Freesurfer software (http://surfer.nmr.mgh.harvard.edu) (Fischl et al., 2002, 2004) was used for cortical surface reconstruction and parcellation. ROIs (Figure 1) were chosen based on clusters of significant activation during a visual search task with a facilitatory auditory cue adapted from (Vaina et al., 2010; Calabro et al., 2011).

#### Data simulation

Simulated data consisted of 20, 50, or 80 trials with phase locking between all ROI pairs and 20, 50, or 80 trials with no phase locking. The trials with no phase locking serve as a means for baseline comparison for the USTPL [see section “Uniform Scores Test for Phase Locking (USTPL)] and PBI (see section “Phase Bifurcation Index”) methods. Phase locking was simulated through the following steps. First, we randomly selected, from a uniform circular distribution, a fixed phase angle for each ROI to ensure that all ROIs will be phase locked. Next, in order to perturb the phase angle slightly between trials, we added a zero-centered von-Mises-distributed random angle (Mardia and Jupp, 2000) with a varying concentration parameter κ = (20, 50). We define the von-Mises distribution as follows:

(1) $$f(x|\theta ,\kappa )=\frac{e^{\kappa \;\cos(x-\theta )}}{2\pi I_{0}(\kappa )},$$

where θ is the phase angle at which the distribution is centered, κ is a measure of concentration of the distribution around θ, and *I*_0_ is the modified Bessel function of order 0.

We then produced a 40-Hz sinusoidal time course in each ROI with a phase shift equal to the perturbed phase angle associated with that ROI. This was repeated for each trial and we thus obtain simulated source-space waveforms **x**_*k*_(*t*) for each trial *k*.

**Figure 2:**
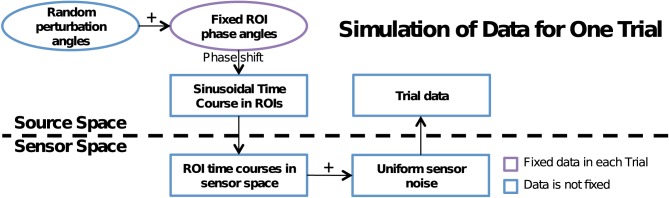
**Flowchart of data simulation.** A dotted line in the middle separates source and sensor space computations. A random perturbation phase angle is added to the fixed phase angle at each ROI to induce a phase shift on the sinusoidal time course of that ROI. The time course is mapped to the sensor space, sensor noise is added and the simulated noisy signals are mapped back to the source space using the MNE linear inverse operator. In trials without phase locking, the fixed ROI phase angle was also randomized.

To accurately model the MEG data measurements as well as to induce cross-talk, we performed a multi-step process summarized in Figure 2. First, simulated data in each ROI, generated in the cortical source space was mapped to the sensor space using the forward operator (gain matrix) **G**, which was computed using a single-compartment boundary-element model with the shape of the inner skull surface extracted from the MRI data of the subject (Hamalainen and Sarvas, 1989). Next, we added in sensor noise **n**_*k*_(*t*) to each trial. This was obtained from the pre-stimulus baseline data of the experiment described in Vaina et al. (2010). We first computed an estimate for the noise covariance matrix from these data and then used this matrix to obtain spatially colored noise from Gaussian noise with unit variance and zero mean, independent across sensors. As a result we obtain the noisy sensor-space signals:

(2) $$y_{k}(t)=Gx_{k}(t)+n_{k}(t)$$

As an inverse solution we employed the cortically constrained, depth-weighted L2 MNE (Hämäläinen and Ilmoniemi, 1994; Dale et al., 2000; Lin et al., 2006). These computations were performed using the MNE software (http://www.nmr.mgh.harvard.edu/martinos/userInfo/data/sofMNE.php), which amounted to multiplying **y**_*k*_(*t*) with a linear inverse operator **W**:

(3) $${x^{\prime }}_{k}(t)=Wy_{k}(t)=W(Gx_{k}(t)+n_{k}(t))$$

In the computation of **W**, we constrained the source orientations to be normal to the cortex and used the same noise-covariance matrix as for generating **n**_*k*_(*t*). For each we then computed an average MNE waveform for each ROI. To avoid signal cancellation in the averaging, we calculated the principal direction of the cortical surface normals within each ROI and inverted the sign of the waveform at the given vertex if the surface normal at this vertex pointed to a direction opposite to the principal surface normal direction.

In trials with no phase locking, the phase angle associated with each ROI was randomized on a trial-by-trial basis. This ensured an equal signal amplitude without any phase-synchrony.

#### Wavelet filtering for phase locking

We extracted phase time courses in each ROI for a particular frequency by wavelet filtering. The trial-by-trial ROI time courses were decomposed into complex time-frequency coefficients through the Morlet wavelet transform (Lin et al., 2004) with envelope bandwidth of 1/5 octaves. The time courses in each ROI were filtered with a Morlet wavelet at 40 Hz at 0.5 s into the stimulus to obtain a complex time and frequency filter coefficient for each trial.

#### Computation of receiver operating characteristic curves

A Receiver Operating Characteristic (ROC) curve is a representation of the error of classification over varying thresholds (Green and Swets, 1974). It is a plot of the true positive rate (TPR) vs. the false positive rate (FPR) of a detector, which in this case is the detection of significant phase locking. This method is ideal to compare the four phase locking methods since the PLB and PLI statistics will not provide a significance level directly.

For each set of parameters for each method, we repeat the data simulation of section “Data Simulation” 20 times to obtain a set of statistics from which to construct the ROC curve. We computed one set of 10 simulations to produce positives, with a set of phase-locked trials. Another set of 10 simulations produced negatives, with no phase-locked trials. For each set of parameters and each iteration of the methodology from section “Data Simulation”, we obtained a single statistic at 0.5 s into the sample (see section “Wavelet Filtering for Phase Locking”) from every ROI comparison. Since there are 22 ROIs, each simulation iteration produced 231 comparisons. Combining across all simulations, we obtained 2310 positives and 2310 negatives total.

We sorted the statistics from all iterations by their statistical value and used each as a threshold, such that a value that is equal to or more significant than the current threshold was labeled a positive and ones that are lower were labeled a negative. Thus, we computed two values that characterize the performance of each phase locking detection method: The TPR, which is the rate at which positives were correctly classified as positives, and the FPR, which is the rate at which negatives were incorrectly classified as positives. The TPR and FPR were computed as TPR = TP/P and FPR = FP/N, respectively. Here, TP is the number of positives accurately evaluated as significant and P is the total number of true positives while FP is the number of false positives incorrectly classified as significant and N is the total number of true negatives. For every threshold, we obtained a single point on the ROC curve, using the TPR and FPR. Thereafter, we sorted the values according to the threshold and connected the ordered points to obtain the final ROC curve.

We characterized each ROC curve with the area under the curve (AUC). For a completely random assignment to positive and negative detection, the ROC curve is a line TPR = FPR resulting in AUC = 0.5. When TPR > FPR, the AUC increases reaching AUC = 1 when TPR = 1 and FPR = 0.

Error bounds were computed from the above procedure computing the AUC over 5 sets of 4 iterations (using the 20 trials) to generate 5 separate ROC curves for each set of parameters. The mean is reported as the AUC value and the standard deviation is reported as the interval.

#### Computation of error rates

When a statistical significance is readily available, in the case of PLV and USTPL, we may compute an error rate. Using a significance level at 0.05 with and without Bonferroni correction, we computed the error as:

(4) Error=(TP+TN)/(P+N)

This is the number of correctly assigned positive and negative phase locking detections divided by the total number of tests.

### Phase locking methods

We illustrate with an example the shortcomings of the traditional phase locking method, which tests for similarity of trial-by-trial signal phase angle differences between two sources. If the difference of the phase angles is uniformly distributed across trials, the traditional method assumes that there is no phase locking, since there is no coherence between the phase angles. However, crosstalk may induce a spurious coherence of phase angles. Our method extends the traditional phase locking method by testing the phase angle differences tested against an empirical null distribution that we can sample from the pre-stimulus interval.

#### Traditional phase locking

**Figure 3:**
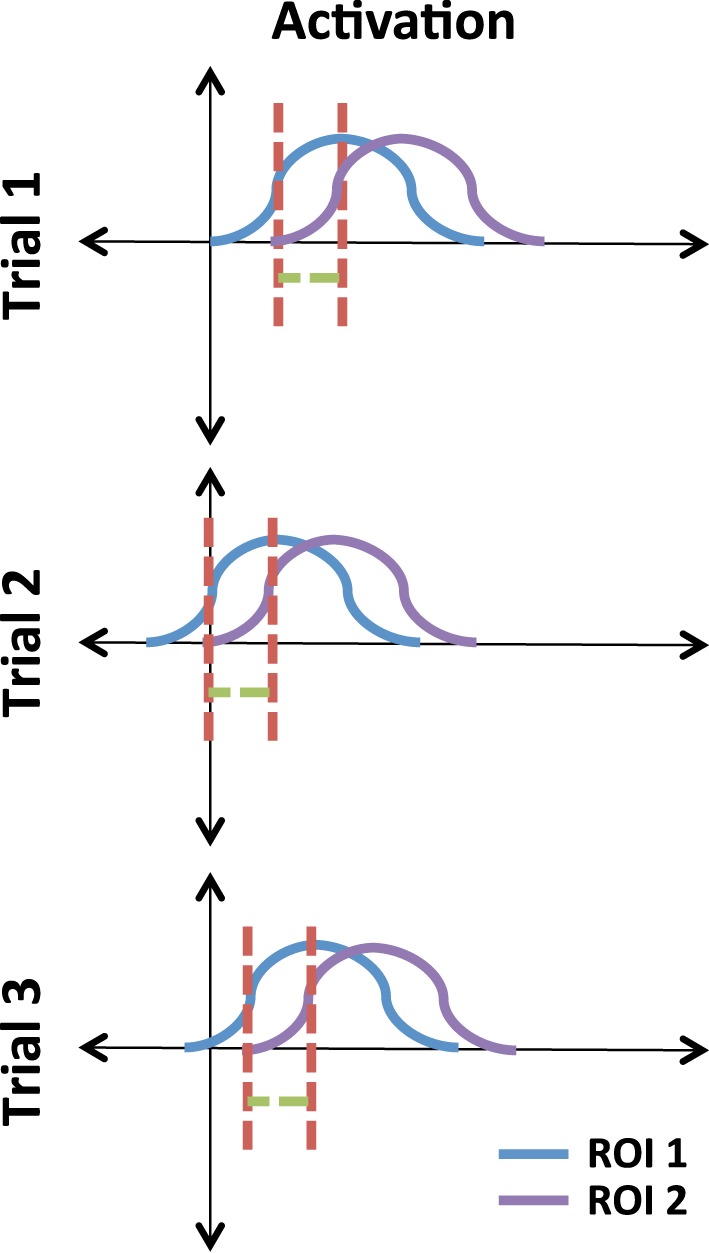
**Illustrative example of phase-locked signals.** The red line is a reference relative to the peak of the signals. The green line illustrates a consistent lag between ROI 1 and ROI 2 across trials.

The concept of phase locking lies in the idea of phase as a shift in a signal. Figure 3 illustrates this using three pairs of time courses. Note that the signals in ROI 1 and ROI 2 occur at random times but their relative phase lag is consistent. We can use circular statistics to assess whether this lag, across trials, is consistent.

To compute the phase lag at different frequencies, we utilize wavelet filtering that produces phase estimates for each time point and for desired frequencies, see section “Wavelet Filtering for Phase Locking”. We can then calculate the trial-by-trial phase differences between the two ROIs as a function of time and frequency.

The traditional phase locking methodology assumes that the two time courses, if not phase locked are completely independent, and thus the phase angle differences will be uniformly random. Therefore, one can apply a test of uniformity to assess whether the distribution of phase angle differences is indeed uniform. The most common uniformity test is the Rayleigh test for circular uniformity (Jervis et al., 1983; Tallon-Baudry et al., 1996; Lin et al., 2004). The Rayleigh test considers each phase angle as a vector with unit length and angle equal to the test angle. First, the phase angle difference between two ROIs is computed for each trial and the corresponding unit vector is found. Then, the vectors are averaged across trials to produce a single averaged vector. The magnitude of the average vector is the Phase Locking Value (PLV). This value ranges from zero to one, corresponding to non-existent and complete phase locking, respectively. When the number of phase angles averaged is large (*n* > 50), the significance of the phase locking value can be approximated (Lin et al., 2004) by:

(5) $${\text{P}}_{\text{PLV}}\sim \text{exp}(-\text{PLV}).$$

#### Crosstalk and phase locking

While the traditional phase locking method works well for two sources whose time courses can be determined without interference, the cortical source estimates in neighboring regions, computed from MEG sensor signals, are prone to crosstalk (Liu et al., 2002) leading to false-positive phase locking detection as discussed in section “Data Simulation.”

To demonstrate this, we drew the phase angle differences as samples from a von Mises distribution. We used this distribution since it allowed us to control sampling from a unimodal distribution with a specified mean and variance, through θ and κ, respectively. As κ increases, the probability of sampling closer to θ increases. The von-Mises distribution approximates the circular normal distribution, which is analogous to the normal distribution for circular statistics (Mardia and Jupp, 2000).

**Figure 4:**
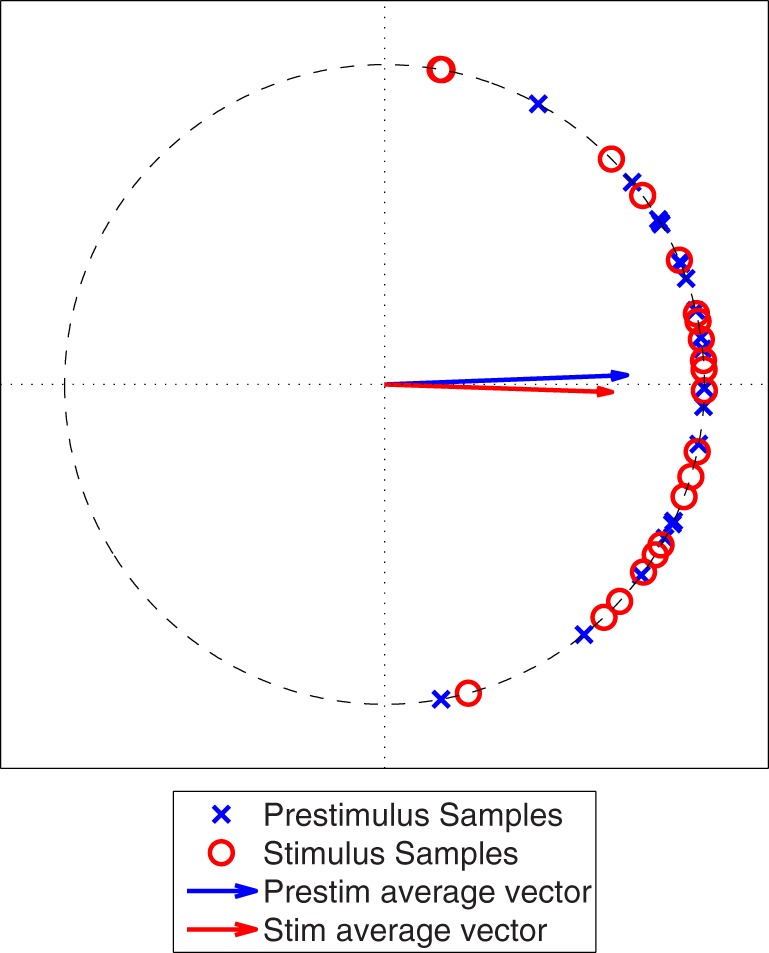
**Simulated phase angle differences for a poststimulus period (Stim) and a prestimulus period (Prestim), both generated from a von Mises distribution with θ equal to zero and κ equal to 4.** The vectors represent the vector averages of the data points corresponding to each population of phase angle differences.

We generated two sets of 20 samples from a von Mises distribution (θ = 0, κ = 4) to simulate sampling from a pre-stimulus interval and a post-stimulus interval where there is no interaction between the two ROIs, see Figure 4. A Rayleigh test applied to the PLV calculated from the stimulus samples showed that this PLV is significant (α < 0.05). However, the distribution in the pre-stimulus interval is similar to the post-stimulus interval, implying that there is no change in phase locking between the pre- and post-stimulus intervals. Therefore, it is necessary to perform a statistical test that compares the two distributions in a fast, efficient manner.

**Figure 5:**
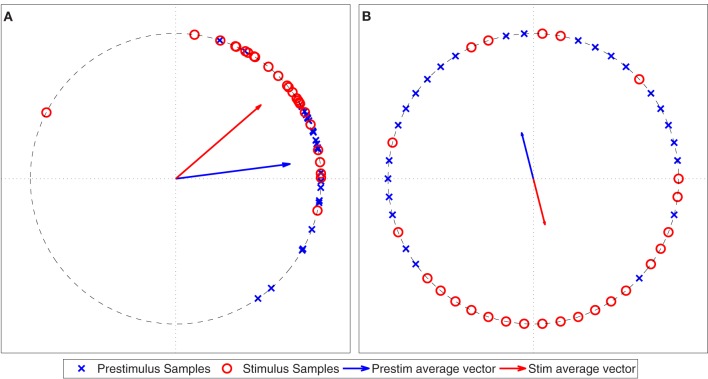
**(A)** Simulated phase angle differences from von Mises distributions for a post-stimulus period (θ = π/4, κ = 4) and a pre-stimulus period (θ = 0, κ = 4). **(B)** Same data redistributed by their corresponding circular rank statistic. The vectors represent the vector averages of the data points corresponding to each population of circular ranks.

While one could assume that it should be sufficient to compare the average vector magnitudes via permutation tests or bootstrap resampling, these procedures computationally taxing procedures and may also lose useful phase information, as illustrated in Figure 5A. The PLV's are similar but one can see that there is a phase shift between the two. Specifically, the pre-stimulus phase angle differences are distributed near zero whereas the post-stimulus phase angle samples are close to π/4. This suggests the possibility of a communication lag, or a potential change in the delay between information passed through ROI 1 and ROI 2 from the pre- to post-stimulus period. To account for these differences, we propose a statistical test that compares two distributions of the phase angles.

#### Uniform scores test for phase locking (USTPL)

**Figure 6:**
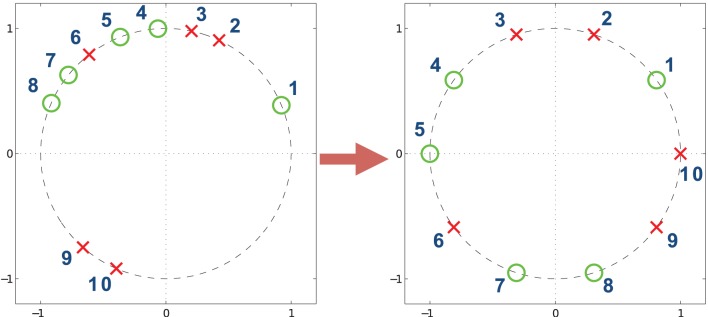
**An example of circular rank statistics.** The circle on the left shows points before circular ranking whereas on the right the points are ranked and repositioned. The numbers indicate the rankings of the points. The repositioned points are equally spaced around the unit circle and the order of the classification of the red and green points is preserved.

We propose the Uniform Scores Test (UST) (Mardia and Jupp, 2000) as a method to compare two phase angle distributions. In this test, the samples of phase angles from the two distributions are linearly ranked jointly so the rank 1 data point will be closest to zero degrees in the positive direction, the rank 2 data point will be the second closest, while the last data point will be furthest from 0, or closest to 2π. The ranked phase angles are then evenly positioned around the unit circle; the corresponding polar angles are then called the circular rank statistics (Mardia and Jupp, 2000). This is illustrated in Figure 6.

Using the ranked phase angles, we next compute a statistic similar to the phase locking value:

(6) $$R_{k}^{2}={\left(\sum_{i=1}^{n_{k}}\cos\beta_{i}^{\left(k\right)}\right)}^{2}+{\left(\sum_{i=\;1}^{n_{k}}\sin\beta_{i}^{\left(k\right)}\right)}^{2}$$

where *n*_*k*_ is the size of phase angle population considered, *k*, (in this case, we can choose the post-stimulus population) and β^(*k*)^_*i*_ is the circular rank statistic of the *i*th element of population *k*. To take advantage of asymptotic bounds for large sample sizes, we normalize *R*_*k*_ to create a new statistic:

(7) $$R_{k}^{*}=\frac{2(n-1)R_{k}}{n_{1}n_{2}}$$

As the total number of samples grows large (*n*_1_ + *n*_2_ = *n* > 40), the null distribution R^*^_k_ approaches a χ^2^ distribution with two degrees of freedom (Mardia and Jupp, 2000).

For demonstrative purposes, we apply USTPL's ranking method in Figure 5A to obtain Figure 5B. The redistribution of angles clearly illustrates the separation of the two distributions by phase, and thus the difference between the prestimulus and poststimulus intervals.

#### USTPL for comparing stimulus conditions

Since the UST is a comparative statistical test, it can be used to compare any pair of phase angle populations, e.g., two stimulus conditions A and B. Instead of employing UST between the post-stimulus and pre-stimulus periods, we can compute it between the conditions A and B. One drawback is that, although this test will allow detection of a significant phase locking difference between A and B, the test alone cannot tell which is stronger or whether the phase difference has changed between conditions A and B.

#### Phase lag index

A recently proposed approach to solve the cross-talk problem uses a statistic known as the PLI (Stam et al., 2007; Vinck et al., 2011). In this method, it is assumed that a common source of noise or cross-talk between two sources is associated with a phase angle of either 0 or π, the latter corresponding to a change in polarity only. PLI, therefore, sets out to measures the asymmetry between the number of phase angles whose sines are positive and negative. The PLI statistic is defined as:

(8) PLI=|〈sign[sinθ]〉|,

where θ is the phase angle difference. If there is a bias of phase angle differences toward either side, the PLI will deviate from zero, which indicates significance. In our simulations (see section Crosstalk and Phase Locking”), we did not discriminate between leading or lagging phases, therefore, in our analysis, we do not differentiate between a resulting mean sign that is above or below zero. Therefore, we take the magnitude of the mean sign as the PLI.

Vinck et al. (2011) introduce a weighting term to form a weighted PLI (WPLI):

(9) $$\text{WPLI}=\frac{\left|\langle \left|ℑ\left\{X\right\}\right|\text{sign}\left[\sin\theta \right]\rangle \right|}{\langle \left|ℑ\left\{X\right\}\right|\rangle },$$

with ℑ{X}=A sinθ, where *A* is the magnitude of the signal. Thus, for signals that are small, the PLI score will not be as strongly affected. In our simulation the signal amplitudes are held constant between the baseline and the stimulus, and differences between PLI and WPLI would occur only due to the presence of noise. Since the signal amplitude is held constant in our simulations and that amplitude measures are not incorporated into other methods, we use Stam's formulation of PLI to compare against the other methods.

#### Phase bifurcation index

We will also consider another method to that uses a similar concept of a phase angle distribution comparison, is the PBI proposed by Busch et al. (2009) defined as:

(10) $$\text{PBI}=({\text{PLV}}_{1}-{\text{PLV}}_{\text{all}})\times ({\text{PLV}}_{2}-{\text{PLV}}_{\text{all}})$$

where PLV_1_, PLV_2_, and PLV_all_ are the phase locking values from distribution 1, 2, and the combined population, respectively. The phase locking value of the individual distributions (PLV_1_, PLV_2_) are calculated by applying the method in section “Traditional Phase Locking” to the subset of trials from which the distribution (either 1 or 2, such as the prestimulus or poststimulus) is sampled. To compute PLV_all_, the method should be applied to the grouped trials from 1 and 2. Each of the differences in Equation (10) compares each population to the combined one. Due to the multiplication of the two differences, the PBI statistic will be significant if both populations are significantly different from the combined population.

#### Obtaining statistical significance measures

In the above methodologies, only PLV and USTPL provide a direct method for obtaining a statistical significance level. In order to obtain statistical significance with PLI or PBI, non-parametric methods may be used. Since we will be performing a comparison between two populations, a simple permutation test should act as an appropriate means to compute a difference. For PLI, we can do so by computing the PBI during the stimulus and nonstimulus interval over a number of iterations while permuting between the two. However, this means that the statistic has to be computed a large number of times. A conservative number would be to perform 1000 permutations. For PBI, samples for PLV_1_ and PLV_2_ can be permuted between each other, but this requires recomputing PLV for each 1000 times.

Table 1 describes the time required to compute the significance of a single time-frequency datapoint tested on a Macbook Pro (version 8,1, Late 2011) with 100 trials. As we sample a larger number of time-frequency points, the computation time increases linearly, and thus multiplicatively if computed across a full grid of time and frequency coordinates as we increase the samples on either axis. It is clear that for such scenarios, it would be computationally expensive to compute the bootstrap for PLI or PBI.

**Table 1:** **Time for computing a single significance statistic**.

**Method**	**Computation time (s)**
USTPL	0.01538 ± 0.00014
PLI	0.01520 ± 0.00014
PBI	15.2181 ± 0.1416
PLV	15.2199 ± 0.1415

This table illustrates the difference in time for computing the PLI and PBI statistics, requiring expensive non-parametric techniques (in this case permutation testing). USTPL and PLV can be computed in milliseconds whereas it takes seconds for PLI or PBI for obtaining a single significance statistic. Calculations were performed over 50 trials for each the stimulus and prestimulus periods on a Macbook Pro (version 8,1, Late 2011). The times are reported as a mean ± the standard error.

## Results

We first discuss the performance of our proposed method, USTPL, compared to PLI, PBI, and PLV in detecting phase locking across trials at a particular time slice with help of ROC curves (Green and Swets, 1974). Second, we compare the error rates of USTPL and PLV for a given statistical significance level. Since PLI and PBI do not produce significance levels directly, we do not consider them in our comparison.

### Detection performance

**Figure 7:**
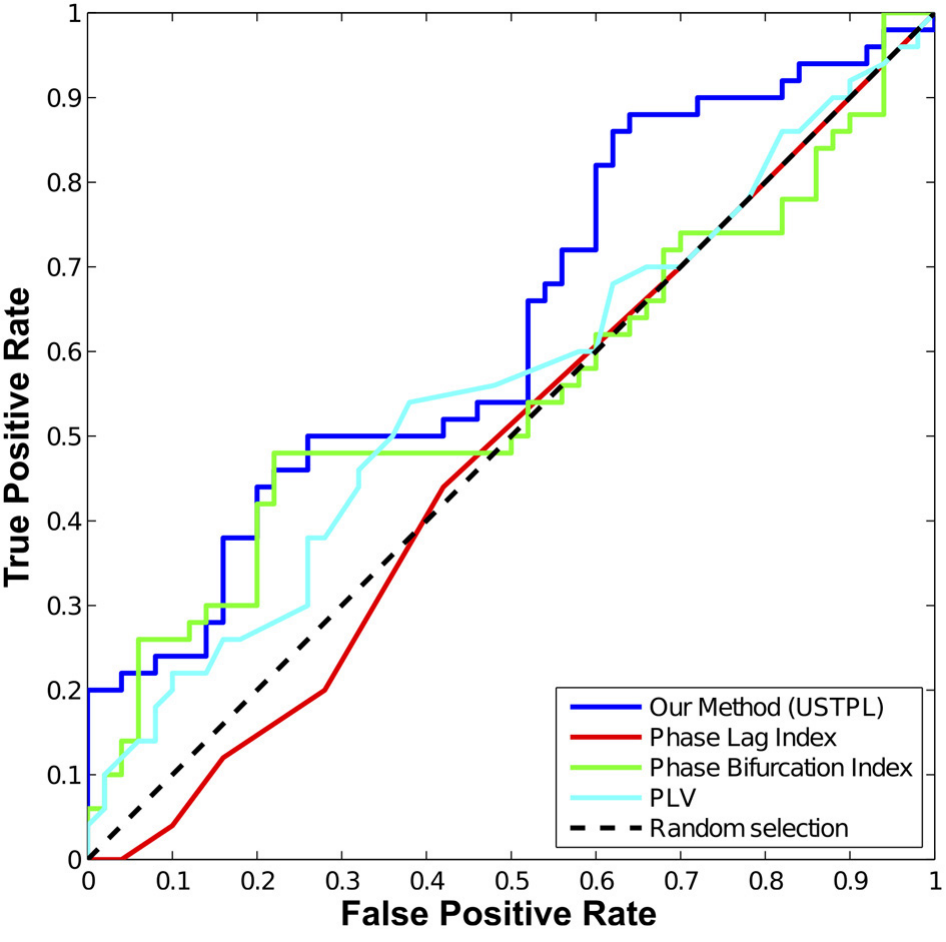
**ROC curve comparing the four statistics at a mean noise level equal to approximately 0.1% of the signal level, κ = 50, number of trials (*n*) = 20.** USTPL (blue) has more detection power than PLV (cyan), PLI (red), and PBI (green).

The comparison of the ROCs of the four methods shown in Figure 7 indicates that, overall, USTPL (shown in blue) has a higher true-positive rate than the other three methods in the condition of a mean noise level equal to 0.1% of the signal level, random angle concentration parameter κ = 50, number of trials *n* = 20. Although USTPL's ROC curve approaches a TPR of 50% as the FPR approaches 50%, this is not likely to be inherent to the method but is unique to this particular simulation at these parameter values. Table 2 summarizes the AUC values across different conditions.

**Table 2:** **Performance of all three methods for various noise levels, number of trials (*n*), and concentration factors (κ)**.

**Noise lv:**	***n* = 50, κ = 50**		***n* = 20, κ = 50**	
	**0.10%**	**1.00%**	**0.10%**	**1.00%**
USTPL	0.5917 ± 0.0839	0.4962 ± 0.0117	0.5900 ± 0.0387	0.4974 ± 0.0233
PLI	0.6460 ± 0.0744	0.5119 ± 0.0203	0.5025 ± 0.0271	0.4983 ± 0.0139
PBI	0.5371 ± 0.0233	0.4938 ± 0.0128	0.5246 ± 0.0282	0.5144 ± 0.0174
PLV	0.5032 ± 0.0164	0.4966 ± 0.0126	0.5198 ± 0.0229	0.4840 ± 0.0037
**Noise lv:**	***n* = 80, κ = 50**		***n* = 50, κ = 20**	
	**0.10%**	**1.00%**	**0.10%**	**1.00%**
USTPL	0.5989 ± 0.0639	0.5052 ± 0.0067	0.4996 ± 0.0187	0.5051 ± 0.0156
PLI	0.6693 ± 0.0663	0.4985 ± 0.0118	0.4991 ± 0.0115	0.5106 ± 0.0113
PBI	0.5190 ± 0.0072	0.5100 ± 0.0108	0.5101 ± 0.0125	0.5048 ± 0.0126
PLV	0.5038 ± 0.0173	0.4914 ± 0.0163	0.5051 ± 0.0199	0.5116 ± 0.0146

The numbers are reported as the mean ±1 standard deviation over 5 simulation repetitions (section “Computation of Receiver Operating Characteristic Curves”).

As κ decreases, we find a significant drop in performance of all methods. As number of trials (*n*) decreases significance strength decreases most for PLI. Signal level has a significant effect on detection performance as well.

### Significance level error rate

**Figure 8:**
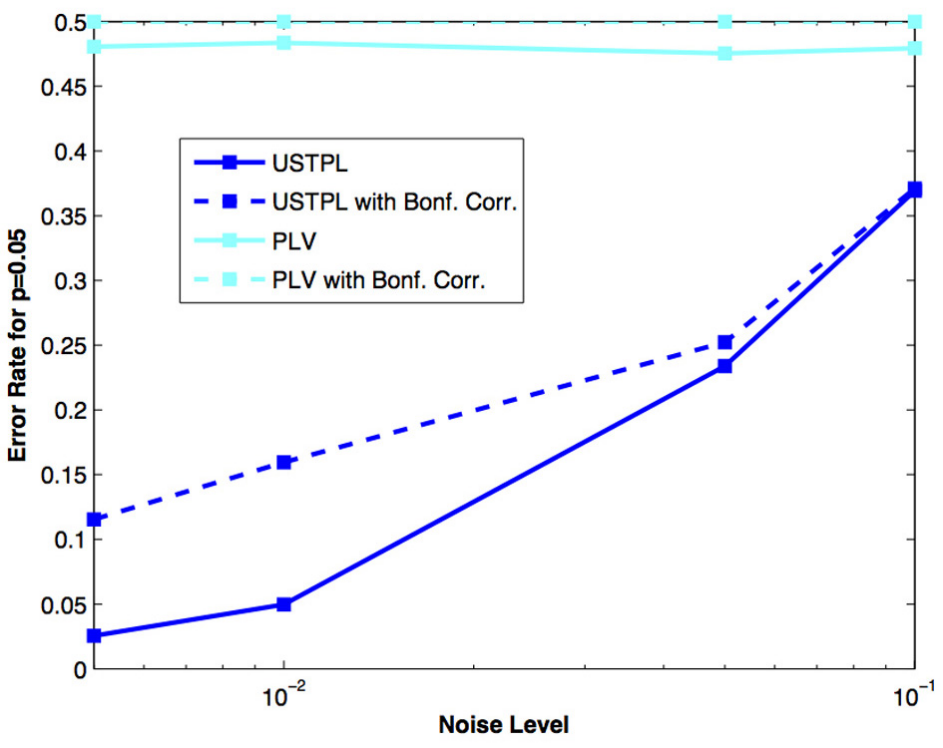
**Comparison of error rates at *p* = 0.05 with and without Bonferroni correction between PLV and USTPL methods.** Dotted lines indicate error rates at Bonferroni-corrected significance values. USTPL is significantly lower than PLV at all tested noise levels.

Figure 8 shows that for a significance level threshold *p* = 0.05, we obtain lower error rates when using USTPL compared to PLV. Interestingly, the Bonferroni-corrected significance levels cause an increase in the error rates. This is understandable because the correction will increase the significance of the threshold, and thus true positives with low-significances will be incorrectly classified as false. However, this correction is appropriate in experimental situations when true positives are fewer and false positives are essential to be removed.

It is interesting that in this simulation, the traditional phase locking method has nearly no detection power. However, this does not mean it has no use in MEG. Due to the closeness of ROIs on the brain in this simulation, the cross-talk is significant. Thus, leakage into neighboring ROIs is likely to induce a detectable bias to the phase differences between these neighboring ROIs. In addition, our simulations were conducted at a single frequency. Therefore, all sources, even in the baseline, will generate signals at this frequency and thus induce cross-talk. In a more realistic situation, there would be activations at different frequencies and thus there will be a smaller set of areas where activation occurs at a certain frequency.

## Discussion

This paper provides a novel statistical method to compare the phase angle differences between two ROIs to an empirical null distribution using the uniform scores test, producing a statistical significance value. We compared this new method (USTPL) to three existing methods, PLV, PLI, and PBI. Unlike PLI or PBI, USTPL does not require computationally expensive non-parametric statistical methods significance levels. Thus this method can be easily used for testing many frequencies and time points between a large number of ROIs, or for computing statistics across the cortical surface as in Lin et al. (2004) for PLV.

In our simulation, we were able to detect phase locking when the concentration parameter was high and noise was low using PLI, PBI, and our method, USTPL. However, the performance of PBI was still lower than PLI and USTPL in these situations. PLI, on average, slightly outperformed USTPL when the number of trials was large (*n* = 50, 80), though the difference was not significant. However, USTPL maintained its performance level when the number of trials was decreased to be below the asymptotic limit of its significance calculation. This shows that USTPL is robust when limited trials are available as opposed to PLI, most likely due to the information lost in PLI due to its capturing of only sign changes weighted by signal magnitudes whereas USTPL utilizes the distribution of phase angles for differentiating the baseline from the test population. However, there is a slight advantage to using PLI when the number of trials is large and, like in our controlled experiment, the baseline contains phase locking due to instantaneous effects, such as crosstalk.

In situations where the prestimulus period is random, while the post-stimulus period is phase-locked, we would expect that PLV, a parametric method, would have more statistical power than USTPL, a non-parametric method, since the assumption of a uniform phase distribution holds. However, this is often not the case since, due to source modeling or closely spaced sources, any correlations would break the assumption of phase distribution uniformity.

Another caveat of our proposed method is that we lose information about differences between phase angle populations. For example, if we are comparing two stimulus conditions with USTPL, this test alone does not tell us which condition had stronger phase locking, or whether one condition leads to a greater phase lag. When comparing against a baseline, we assume that there is no phase locking within the baseline period. If there is suspected phase locking in the baseline, other measures may be necessary to supplement differences found in USTPL. However, the USTPL method will provide knowledge of those frequency and time points amongst ROIs that have significantly different phase locking and will thus provide a smaller set of connections to investigate with more scrutiny.

Due to the bivariate nature of phase locking analysis the four measures discussed in this paper do not differentiate between direct communication between two ROIs and having a third source driving both. Methods have been developed to consider the multivariate network and finding partial phase locking (Schelter et al., 2006; Cadieu and Koepsell, 2010; Canolty et al., 2012). In the future we plan to extend the USTPL model by incorporating these multivariate methods.

### Conflict of interest statement

The authors declare that the research was conducted in the absence of any commercial or financial relationships that could be construed as a potential conflict of interest.

## References

[B1] Busch N. A. Dubois J. Vanrullen R. (2009). The phase of ongoing EEG oscillations predicts visual perception. J. Neurosci. 29, 7869–7876 10.1523/JNEUROSCI.0113-09.2009 19535598 PMC6665641

[B2] Cadieu C. F. Koepsell K. (2010). Phase coupling estimation from multivariate phase statistics. Neural Comput. 22, 3107–3126

[B3] Calabro F. J. Soto-Faraco S. Vaina L. M. (2011). Acoustic facilitation of object movement detection during self-motion. Proc. Biol. Sci. 278, 2840–2847 10.1098/rspb.2010.2757 21307050 PMC3145189

[B4] Canolty R. T. Cadieu C. F. Koepsell K. Ganguly K. Knight R. T. Carmena J. M. (2012). Detecting event-related changes of multivariate phase coupling in dynamic brain networks. J. Neurophysiol. 107, 2020–2031 10.1152/jn.00610.2011 22236706 PMC3331660

[B5] Dale A. M. Fischl B. Sereno M. I. (1999). Cortical surface-based analysis: I. Segmentation and surface reconstruction. Neuroimage 9, 179–194 10.1006/nimg.1998.0395 9931268

[B6] Dale A. M. Liu A. K. Fischl B. R. Buckner R. L. Belliveau J. W. Lewine J. D. (2000). Dynamic statistical parametric mapping: combining fMRI and MEG for high-resolution imaging of cortical activity. Neuron 26, 55–67 10.1016/S0896-6273(00)81138-1 10798392

[B7] Dale A. M. Sereno M. I. (1993). Improved localization of cortical activity by combining EEG and MEG with MRI cortical surface reconstruction: a linear approach. J. Cogn. Neurosci. 5, 162–176 10.1162/jocn.1993.5.2.162 23972151

[B8] Fischl B. Liu A. Dale A. M. (2001). Automated manifold surgery: constructing geometrically accurate and topologically correct models of the human cerebral cortex. IEEE Trans. Med. Imaging 20, 70–80 10.1109/42.906426 11293693

[B9] Fischl B. Salat D. H. Busa E. Albert M. Dieterich M. Haselgrove C. (2002). Whole brain segmentation: automated labeling of neuroanatomical structures in the human brain. Neuron 33, 341–355 10.1016/S0896-6273(02)00569-X 11832223

[B10] Fischl B. Sereno M. I. Dale A. M. (1999a). Cortical surface-based analysis: II: inflation, flattening, and a surface-based coordinate system. Neuroimage 9, 195–207 10.1006/nimg.1998.0396 9931269

[B11] Fischl B. Sereno M. I. Tootell R. B. H. Dale A. M. (1999b). High-resolution intersubject averaging and a coordinate system for the cortical surface. Hum. Brain Mapp. 8, 272–284 10.1002/(SICI)1097-0193(1999)8:4<272::AID-HBM10>3.0.CO;2-4 10619420 PMC6873338

[B12] Fischl B. van der Kouwe A. Destrieux C. Halgren E. Ségonne F. Salat D. H. (2004). Automatically parcellating the human cerebral cortex. Cereb. Cortex 14, 11–22 10.1093/cercor/bhg087 14654453

[B13] Gloor P. (1975). Contributions of electroencephalography and electrocorticography to the neurosurgical treatment of the epilepsies. Adv. Neurol. 8, 59–105 804238

[B14] Green D. M. Swets J. A. (1974). Signal Detection Theory and Psychophysics. Oxford, England: Robert, E. Krieger.

[B15] Hämäläinen M. Hari R. Ilmoniemi R. J. Knuutila J. Lounasmaa O. V. (1993). Magnetoencephalography-theory, instrumentation, and applications to noninvasive studies of the working human brain. Rev. Mod. Phys. 65, 413–497

[B16] Hämäläinen M. Ilmoniemi R. (1994). Interpreting magnetic fields of the brain: minimum norm estimates. Med. Biol. Eng. Comput. 32, 35–42 8182960 10.1007/BF02512476

[B17] Hamalainen M. S. Sarvas J. (1989). Realistic conductivity geometry model of the human head for interpretation of neuromagnetic data. IEEE Trans. Biomed. Eng. 36, 165–171 10.1109/10.16463 2917762

[B18] Hui H. B. Pantazis D. Bressler S. L. Leahy R. M. (2010). Identifying true cortical interactions in MEG using the nulling beamformer. Neuroimage 49, 3161–3174 10.1016/j.neuroimage.2009.10.078 19896541 PMC2818446

[B19] Jervis B. W. Nichols M. J. Johnson T. E. Allen E. Hudson N. R. (1983). A fundamental investigation of the composition of auditory evoked potentials. IEEE Trans. Biomed. Eng. 1, 43–50 6826185 10.1109/tbme.1983.325165

[B20] Keene D. L. Whiting S. Ventureyra E. C. (2000). Electrocorticography. Epileptic Disord. 2, 57–63 10937174

[B21] Lin F.-H. Belliveau J. W. Dale A. M. Hämäläinen M. S. (2006). Distributed current estimates using cortical orientation constraints. Hum. Brain Mapp. 27, 1–13 10.1002/hbm.20155 16082624 PMC6871274

[B22] Lin F.-H. Witzel T. Hämäläinen M. S. Dale A. M. Belliveau J. W. Stufflebeam S. M. (2004). Spectral spatiotemporal imaging of cortical oscillations and interactions in the human brain. Neuroimage 23, 582–595 10.1016/j.neuroimage.2004.04.027 15488408 PMC2884198

[B23] Liu A. K. Dale A. M. Belliveau J. W. (2002). Monte Carlo simulation studies of EEG and MEG localization accuracy. Hum. Brain Mapp. 16, 47–62 10.1002/hbm.10024 11870926 PMC6871820

[B24] Mardia K. V. Jupp P. E. (2000). Directional Statistics. Chichester: John Wiley and Sons.

[B25] Rana K. Vaina L. M. Hämäläinen M. (2011). A method for reducing cross-talk in MEG data with subspace suppression and the nulling beamformer. Front. Neuroinform. 10.3389/conf.fninf.2011.08.00019

[B26] Robinson S. E. Vrba J. (1999). Functional neuroimaging by synthetic aperture magnetometry (SAM), in Recent Advances in Biomagnetism, eds Yoshimoto T. Kotani M. Kuriki S. Karibe H. Nakasato N. (Sendai: Tohuku University Press), 302–305

[B27] Schelter B. Winterhalder M. Dahlhaus R. Kurths J. Timmer J. (2006). Partial phase synchronization for multivariate synchronizing systems. Phys. Rev. Lett. 96, 208103 10.1103/PhysRevLett.96.208103 16803212

[B28] Schoffelen J.-M. Gross J. (2009). Source connectivity analysis with MEG and EEG. Hum. Brain Mapp. 30, 1857–1865 10.1002/hbm.20745 19235884 PMC6870611

[B29] Stam C. J. Nolte G. Daffertshofer A. (2007). Phase lag index: assessment of functional connectivity from multi channel EEG and MEG with diminished bias from common sources. Hum. Brain Mapp. 28, 1178–1193 10.1002/hbm.20346 17266107 PMC6871367

[B30] Tallon-Baudry C. Bertrand O. Delpuech C. Pernier J. (1996). Stimulus specificity of phase-locked and non-phase-locked 40 Hz visual responses in human. J. Neurosci. 16, 4240–4249 8753885 10.1523/JNEUROSCI.16-13-04240.1996 PMC6579008

[B31] Tallon-Baudry C. Bertrand O. Fischer C. (2001). Oscillatory synchrony between human extrastriate areas during visual short-term memory maintenance. J. Neurosci. 21, RC177. 11588207 10.1523/JNEUROSCI.21-20-j0008.2001 PMC6763859

[B32] Vaina L. M. Calabro F. Lin F.-H. Hämäläinen M. S. (2010). Long-range coupling of prefrontal cortex and visual (MT) or polysensory (STP) cortical areas in motion perception. Front. Neurosci. 10.3389/conf.fnins.2010.06.00212

[B33] Van Veen B. D. Van Drongelen W. Yuchtman M. Suzuki A. (1997). Localization of brain electrical activity via linearly constrained minimum variance spatial filtering. IEEE Trans. Biomed. Eng. 44, 867–880 10.1109/10.623056 9282479

[B34] Vinck M. Oostenveld R. Van Wingerden M. Battaglia F. Pennartz C. M. A. (2011). An improved index of phase-synchronization for electrophysiological data in the presence of volume-conduction, noise and sample-size bias. Neuroimage 55, 1548–1565 10.1016/j.neuroimage.2011.01.055 21276857

